# Impact of FDG-PET findings on decisions regarding patient management strategies: a multicenter trial in patients with lung cancer and other types of cancer

**DOI:** 10.1007/s12149-015-0963-9

**Published:** 2015-03-27

**Authors:** Kazuo Kubota, Shinsuke Matsuno, Nobuo Morioka, Shuji Adachi, Mitsuru Koizumi, Hikaru Seto, Motohisa Kojo, Satoshi Nishioka, Michihiko Nishimura, Hiroshi Yamamoto

**Affiliations:** 1 Division of Nuclear Medicine, Department of Radiology, National Center for Global Health and Medicine, 1-21-1 Toyama, Shinjuku-ku, Tokyo, 162-8655 Japan; 2 Department of Radiology, Takinomiya General Hospital, 486 Takinomiya, Ayagawa Town, Ayauta, Kagawa 761-2305 Japan; 3 Department of Radiology, Matsue Red Cross Hospital, 200 Horomachi, Matsue, Shimane 690-0886 Japan; 4 Department of Radiology, Hyogo Cancer Center, 13-70 Kitaojimachi, Akashi, Hyogo 673-0021 Japan; 5 Department of Nuclear Medicine, Cancer Institute Hospital, 3-8-31 Ariake, Koto-ku, Tokyo, 135-0063 Japan; 6 Department of Radiology, Toyama University Hospital, 2630 Sugitani, Toyama, Toyama 930-0152 Japan; 7 Department of Surgery, Ako Central Hospital, 52-6 Somoncho, Ako, Hyogo 678-0241 Japan; 8 Department of Surgery, Ako Hakuhou-kai Hospital, 99 Aza Shinmachi, Kariya, Ako, Hyogo 678-0239 Japan; 9 Department of Internal Medicine and Clinical Laboratory, Koyo Hospital, 40 Tsuhada, Wakayama, Wakayama 640-8315 Japan; 10 Department of Radiology, Sumitomo Hospital, 5-3-20 Nakanoshima, Kita-ku, Osaka, Osaka 530-0005 Japan

**Keywords:** FDG-PET, Patient management strategy, Comparison between pre- and post-test periods, Lung cancer

## Abstract

**Objective:**

To date, numerous studies have been conducted on the diagnostic capabilities of positron emission tomography using [^18^F]-fluorodeoxyglucose (FDG-PET). However, no studies designed to evaluate the influence of FDG-PET on the selection of patient management strategies within the Japanese healthcare system have been reported to date. The aim of the present study was to investigate prospectively the proportion of patients whose management strategies were modified based on FDG-PET findings (strategy modification rate).

**Methods:**

The strategy modification rate was calculated by comparing the patient management strategy (test and treatment plans) after FDG-PET with the strategy before FDG-PET for 560 cancer patients with nine types of cancer (lung cancer, breast cancer, colorectal cancer, head/neck cancer, brain tumor, pancreas cancer, malignant lymphoma, cancer of unknown origin, and melanoma). In addition, the details of the modifications to the patient management strategies were analyzed.

**Results:**

The strategy modification rate for patients with lung cancer was 71.6 % (149 of 208 patients, 95 % confidence interval 65.0–77.7 %), which was higher than previously reported strategy modification rates for lung cancer before and after FDG-PET (25.6 %). The strategy modification rates for patients with cancers other than lung cancer were as follows: breast, 44.4 % (56/126); colorectal, 75.6 % (62/82); head and neck, 65.2 % (15/23); malignant lymphoma, 70.0 % (35/50); pancreas, 85.0 % (17/20); and cancer of unknown origin, 78.0 % (32/41). The mean modification rate (major and minor modifications) of the treatment plans after FDG-PET, relative to the plans before FDG-PET, was 55.4 % (range 44.0–69.2 %), with major modifications pertaining to the treatment plan made in 43.3–68.2 % of the patients based on the objectives of the FDG-PET examination.

**Conclusions:**

The results from this study indicate that FDG-PET can contribute to the modification of management strategies (particularly treatment plans), especially for lung cancer patients but also for patients with other types of cancer.

## Introduction

To evaluate the efficacy of diagnostic imaging, one can assume a hierarchical model consisting of the following: (1) technical performance (can the target abnormality be visualized?), (2) diagnostic performance (is an accurate diagnosis possible?), (3) efficacy for patient management (is the management plan likely to be modified based on an accurate diagnosis?), (4) efficacy for promoting patient’s health (is the patient’s health likely to be improved as a result of the management plan modification?), and (5) social efficacy (is the diagnostic imaging cost-effective?). Evaluating these steps, in this order, is thought to be appropriate when conducting studies to determine the efficacy of diagnostic imaging [1, 2].

A large number of articles and books concerning the diagnostic performance of positron emission tomography using [^18^F]-fluorodeoxyglucose (FDG-PET) are already available worldwide [3–7]. Thus, the most important step in evaluating the efficacy of FDG-PET as a diagnostic imaging modality, at present, is an evaluation of its efficacy for patient management during clinical practice, i.e., an evaluation of whether the patient management strategy is modified based on the FDG-PET findings.

A randomized inter-group comparison and a comparison of patient management plans between pre- and post-diagnostic imaging periods (comparison between pre-test and post-test periods) are now available as two different approaches for evaluating the efficacy of diagnostic imaging for patient management. A randomized inter-group comparison involves two groups (a group receiving the diagnostic imaging and a group not receiving the diagnostic imaging). Using the inter-group comparison study design, it is difficult to assign the subjects (i.e., to select two groups of patients with strictly matched background variables) to two different evaluation groups. A comparison between pre-test and post-test periods is, on the other hand, a more efficient study design because a diversity of illnesses and pathophysiologies can be included by evaluating a single group of patients [8]. All the previous research on this topic has adopted a “comparison compares patient management plans between pre-test and post-test periods” study design [6, 9–27].

The present study was undertaken to analyze the proportion of patients whose management strategies were modified based on findings obtained from the addition of FDG-PET to their existing test menus (the strategy modification rate) using the concept of a comparison between pre-test and post-test periods. This study was initiated as a pre-marketing clinical study and was later modified to become a post-marketing clinical study because the manufacture and distribution of FDG were approved during the study period. Thus, this study was carried out in accordance with both Good Clinical Practice (GCP) guidelines and Good Post-marketing Study Practice (GPSP) guidelines in Japan [28, 29].

## Materials and methods

This study was performed between April 5 and December 28, 2005, as a multicenter open study involving eight medical facilities. The participating facilities and the study organization are shown in the Appendix. Before the study commenced, the study protocol, case report form, informed consent form for patients, as well as other necessary documents, and the appropriateness of the study were reviewed by the Institutional Review Board (IRB) of each participating facility from ethical and scientific points of view. The IRB of each participating facility approved the study.

### Subjects

The study involved patients with one of nine types of cancer (lung cancer, breast cancer, colorectal cancer, head/neck cancer, brain tumor, pancreas cancer, malignant lymphoma, malignant melanoma and cancer of unknown origin). Informed consent was obtained from each patient before the start of the study. Patients were eligible to participate in the study if their management strategy could be evaluated at both the time of study entry and after an FDG-PET examination conducted for one of the following purposes: (1) distinction between malignant and benign lung nodules and diagnosis of lung cancer metastasis/recurrence, (2) distinction between malignant and benign breast tumors and diagnosis of breast cancer metastasis/recurrence, (3) diagnosis of colorectal cancer metastasis/recurrence, (4) diagnosis of head/neck cancer metastasis/recurrence, (5) diagnosis of brain tumor recurrence, (6) distinction between malignant and benign pancreas tumors, (7) malignant lymphoma staging and diagnosis of recurrence, (8) identification of the primary location of cancer of unknown origin, and (9) diagnosis of malignant melanoma metastasis/recurrence.

The exclusion criteria were as follows: (1) pregnant or possibly pregnant women or lactating women, (2) patients who had participated in any other clinical trial involving the use of FDG, (3) patients who had been treated with any other test drug within the 6 months prior to FDG administration, and (4) patients who were judged by the investigators as being inappropriate for inclusion in a study evaluating FDG efficacy and safety.

### Patient enrollment

Written informed consent to participate in the study was obtained from 578 patients. Of these patients, 565 were administered FDG (565/578, 97.8 %) and 13 were not administered FDG (13/578, 2.2 %) for reasons such as withdrawn consent, and others. After FDG administration, the study was not discontinued in any of the patients. Of the 565 patients who were administered FDG, 5 patients were excluded from the analysis because they failed to meet inclusion criteria or because they met exclusion criteria (5/578, 0.9 %). The remaining 560 patients (560/578, 96.9 %) were included in the analysis. Table 1 shows the demographic data of the patients who were analyzed. Overall, 263 males and 297 females with a mean age of 63.1 years (range: 21–89 years) were included in the analysis.

**Table 1:** Demographic data for the patients analyzed

Variable	Category	No. of patients	Percentage (%)
Total		560	
Sex	Male	263	47.0
	Female	297	53.0
Age (years)	20–29	4	0.7
	30–39	12	2.1
	40–49	65	11.6
	50–59	135	24.1
	60–69	145	25.9
	≥70	199	35.5
	<65	297	53.0
	≥65	263	47.0
	Mean ± S.D.^a^	63.1 ± 12.2	
	Range	21–89	
	95 % CI^b^ (two tailed)	62.1–64.2	–
Objectives of FDG-PET	Lung cancer	208	37.1
	Differential diagnosis	83	14.8
	Staging or metastasis/recurrence	125	22.3
	Staging	73	13.0
	Metastasis/recurrence	52	9.3
	Breast cancer	126	22.5
	Differential diagnosis	4	0.7
	Staging or metastasis/recurrence	122	21.8
	Staging	35	6.3
	Metastasis/recurrence	87	15.5
	Colorectal cancer	82	14.6
	Staging	13	2.3
	Metastasis/recurrence	69	12.3
	Head/neck cancer	23	4.1
	Staging	11	2.0
	Metastasis/recurrence	12	2.1
	Malignant lymphoma	50	8.9
	Staging	16	2.9
	Metastasis/recurrence	34	6.1
	Brain tumor	3	0.5
	Pancreas cancer	20	3.6
	Malignant melanoma	7	1.3
	Staging	2	0.4
	Metastasis/recurrence	5	0.9
	Cancer of unknown origin	41	7.3

^a^ Mean ± 1 standard deviation (SD)

^b^ 95 % confidence interval

### PET scan

Each patient received an intravenous injection of 2 mL of FDG (185 MBq at reference time). The FDG was provided by Nihon Medi-Physics Co., Ltd. (Tokyo, Japan) or The Medical and Pharmacological Research Center Foundation (Ishikawa, Japan). Drip infusions of glucose were suspended and the patients were asked not to drink beverages containing alcohol or carbohydrates from 4 h before FDG administration until the end of PET scanning. Before FDG administration, each patient’s blood glucose level was measured to judge the appropriateness of the FDG administration (cutoff blood glucose level: 200 mg/dL). In diabetic patients receiving insulin for blood glucose control, the administration of insulin was suspended during the 4-h period before FDG administration to stabilize the blood glucose level before FDG administration by avoiding a sharp reduction in the blood glucose level.

PET scanning was started approximately 60 min after the FDG administration (mean 57.4 ± 13.7 min). The initial emission scan (2–3 min × 6–9 scans) was followed by a transmission scan. Image reconstruction was performed using a matrix size of 128 × 128, and preprocessing filters, such as Gaussian filters, FORE filters, or ramp filters (filters were not used at some facilities). OSEM filters were used as reconstruction filters. The presence or absence of a preprocessing filter had no effect on the image assessment. The PET/CT camera used for this study was a Discovery ST (GE) (306 patients) or a Biograph LSO Duo (Siemens) (136 patients). The PET camera used was an ECAT EXACT (Siemens) (60 patients) or an ECAT ACCEL (Siemens) (63 patients). In this study, 442 patients (78.2 %) were evaluated using PET/CT and the remaining 123 patients using PET.

### Efficacy evaluations

**Fig. 1:**
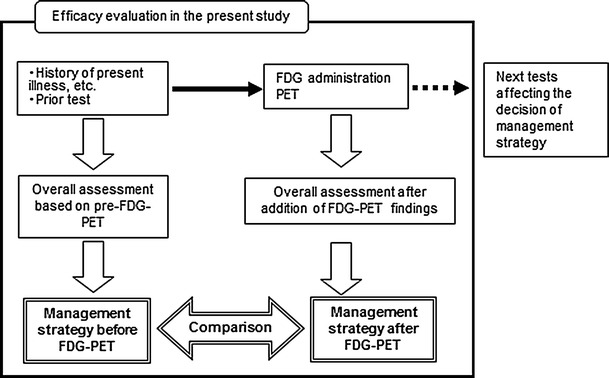
Flow of management strategy evaluation before and after FDG-PET

Figure 1 illustrates the flow of the management strategy evaluation before and after FDG-PET. The percentage of patients whose management strategy as determined before FDG-PET was modified after FDG-PET (strategy modification rate) was analyzed. Investigators at each institution evaluated the management strategy. The parameters/indicators of the management strategy evaluation are shown in Table 2.

**Table 2:** Parameters and definitions of variables for management strategy evaluation

Parameter	Variables
Test plan	(1) Test plan available
	(2) No test plan
	(3) Difficult to devise a test plan at present^a^
Treatment plan	(1) Treatment needed
	(2) Follow-up
	(3) Treatment not needed
	(4) Difficult to devise a treatment plan at present^b^
Presence/absence of additional diagnostic information yielded by FDG-PET	(1) Detection of a new lesion
	(2) Qualitative diagnosis of lesion
	(3) No lesion
	(4) Borderline lesions were visualized
	(5) Others

^a^ Hesitation about an invasive test or impossible to narrow down the test plans

^b^ Vague findings or impossible to choose from multiple treatment plans

The investigators at each institution assessed the overall findings and the patient information available before FDG-PET and decided on the initial management strategy; they then transmitted the records by facsimile to the data center. After the FDG-PET examination, the investigators recorded the overall findings on the addition of the test findings obtained using FDG-PET and checked the presence/absence of additional diagnostic information arising from the FDG-PET examination (Table 2). Based on these overall findings and the additional diagnostic information, a new management strategy was decided (Table 2). The investigators devised the new management strategy based on the FDG-PET findings before any further tests were carried out and then transmitted the records regarding the new management decision by facsimile to the data center. In this manner, the study design minimized the factors affecting the two management strategies adopted before and after the FDG-PET examination, i.e., the modifications to the first management strategy were based only on the FDG-PET findings.

The definitions of management strategy modifications are shown below:

1. The planned tests were modified, supplemented, or skipped after FDG-PET
2. Test planning was difficult before FDG-PET, but became possible after FDG-PET
3. The planned treatments (including treatment methods) were modified, supplemented, or skipped after FDG-PET
4. Treatment planning was difficult before FDG-PET, but became possible after FDG-PET

The modification of the management strategy was investigated by checking the management strategy evaluation sheet filled in by the investigators before and after the FDG-PET examination, with reference to the definition of a modification of the management strategy mentioned above.

Of the variables/indicators analyzed, the primary endpoint was the strategy modification rate for patients with lung cancer, because the number of lung cancer patients who underwent FDG-PET examination was relatively large. The target modification rate was set at 25.6 % because that was the average rate (493/1924 lung cancer patients who underwent FDG-PET) found in 17 reports that we used to estimate the number of subjects needed for our study [5, 9–24]. The secondary endpoint was the percentage of patients with cancer other than lung cancer in whom the management strategy was modified. The target number of subjects with lung cancer for our study was set at 170 to allow estimation of the modification rate of 25.6 % set for the lung cancer patients, with the lower bound of the 95 % confidence interval [CI] being 7 % or less, based on an F distribution. The number of subjects was set at 600 (in total), based on the percentage of each type of disease among all patients who underwent FDG-PET during 1 month in 2004 (in addition to the estimated requirement for 170 lung cancer patients and taking into account the anticipated exclusion of some patients from the analysis).

As a post hoc subanalysis, major changes or minor changes in the treatment plan and changes in the intensity of treatment after FDG-PET, compared with the pre-FDG-PET period, were analyzed. The method used for this subanalysis was based on a report by the National Oncologic PET Registry [30]; the parameters/indicators that were analyzed and the definitions that were used are given in Table 3.

**Table 3:** Criteria for classification of treatment plan modification patterns

Item	Class	Definition	Example
Treatment plan modification	Major 1	Treatment plan category modified	From “difficult to devise a treatment plan” to “treatment needed”
	Major 2	Treatment method modified	From surgery to chemotherapy
	Major 3	Objective of treatment method modified, with no change in treatment method	From curative treatment to palliative treatment
	Minor	Details of treatment method modified, with no change in treatment plan, method, or objective	Operative procedure or drugs for chemotherapy modified
Modification of treatment intensity	Increased	Increase in number of treatment methods	From surgery to surgery and chemotherapy
	Unchanged	No change in number of treatment methods	
	Decreased	Decrease in number of treatment methods	From radiotherapy and chemotherapy to chemotherapy alone

### Evaluation of safety of FDG administration

The parameters/indicators of the safety evaluation included subjective symptoms, objective findings, heart rate, blood pressure, and laboratory parameters such as RBC count, hemoglobin, hematocrit, WBC count, differential leukocyte count (neutrophil, lymphocyte, monocyte, eosinophil, and basophil), platelet count, albumin, Al-P, AST (GOT), ALT (GPT), γ-GTP, LDH, total bilirubin, urea nitrogen, creatinine, Na, K, Cl, urinary protein, urinary glucose, urinary urobilinogen, and urinary occult blood. The safety parameters/indicators were evaluated within 7 days after the FDG administration and were compared with the corresponding values obtained before FDG administration.

### Statistics analysis

Comparisons between the target modification rate (25.6 %) and the modification rate of this study were performed using the *χ* ^2^ test, and differences were considered statistically significant when *p* value was less than 0.05. SAS System ver. 9.2 (SAS Institute Japan, Tokyo, Japan) was used for the statistical analysis.

## Results

### Primary endpoint (lung cancer)

#### Management strategy modification after FDG-PET and calculation of the modification rate (analysis according to patient)

Table 4 shows the management strategy modification rate (percentage of patients whose test plan or treatment plan was modified) and the 95 % CI for patients with lung cancer (*n* = 208). The management strategy modification rate for lung cancer was 71.6 % (149/208 patients, 95 % CI 65.0–77.7 %), which was higher than the target modification rate (25.6 %). The difference was statistically significant (*p* < 0.01). Table 4 also shows management strategy modification rates for lung cancer patients subdivided according to the objective of the diagnostic imaging.

**Table 4:** Management strategy modification rate after FDG-PET (lung cancer, analysis according to patients)

Objective of FDG-PET	No. of patients	Modification rate (%) (no. of modified cases)	95 % CI^a^ (two tailed) for modification rate (%)
Lung cancer	208	71.6 (149)	65.0–77.7
Differential diagnosis	83	88.0 (73)	79.0–94.1
Staging	73	49.3 (36)	37.4–61.3
Metastasis/recurrence diagnosis	52	76.9 (40)	63.2–87.5

^a^ 95 % confidence interval

#### Details of management strategy modification

In an analysis of modifications to the test plan, the most characteristic finding was the modification of the pre-FDG-PET judgment “difficult to devise a test plan at present” (*n* = 59) to the post-FDG-PET judgment “test plan available” (*n* = 25) or “no test plan” (*n* = 33) in 58 (98.3 %) of the 59 patients (Table 5a).

**Table 5:** Modification of lung cancer management strategy based on FDG-PET findings

(a) Test plan						
		After FDG-PET				
		(1) Test plan available		(2) No test plan	(3) Difficult to devise a test plan	Total
		No change in test menu	Change in test menu			
Before FDG-PET	(1) Test plan available	5^a^	10	9	0	24
	(2) No test plan	19		104^a^	2	125
	(3) Difficult to devise a test plan	25		33	1^a^	59
	Total	59		146	3	208

(b) Treatment plan							
		After FDG-PET					
		(1) Treatment needed		(2) Follow-up	(3) Treatment not needed	(4) Difficult to devise a treatment plan	Total
		No change in treatment menu	Change in treatment menu				
Before FDG-PET	(1) Treatment needed	46^b^	19	1	0	2	68
	(2) Follow-up	3		15^b^	1	3	22
	(3) Treatment not needed	0		0	1^b^	0	1
	(4) Difficult to devise a treatment plan	55		40	2	20^b^	117
	Total	123		56	4	25	208

The number of cases to which the test plan was changed before and after FDG-PET was 98 cases. The change rate of test plan by FDG-PET was 47.1 % (98/208)

The number of cases to which the treatment plan was changed before and after FDG-PET was 126 cases. The change rate of treatment plan by FDG-PET was 60.6 % (126/208)

^a^ The number of cases to which a test plan had no change before and after FDG-PET was 110 cases

^b^ The number of cases to which a treatment plan had no change before and after FDG-PET was 82 cases

In an analysis of modifications to the treatment plan, the most characteristic finding was the modification of the pre-FDG-PET judgment “difficult to devise a treatment plan at present” (*n* = 117) to the post-FDG-PET judgment “treatment needed” (*n* = 55), “follow-up needed” (*n* = 40), or “no treatment needed” (*n* = 2) in 97 (82.9 %) of the 117 patients (Table 5b).

### Secondary endpoint (cancers other than lung cancer)

Table 6 shows the management strategy modification rates and their 95 % CIs for patients with cancers other than lung cancer. Because there was little number of cases, brain tumor (*n* = 3) and malignant melanoma (*n* = 7) were eliminated. The modification rate was in the range 44.4–85.0 % for each type of cancer.

**Table 6:** Management strategy modification rate based on FDG-PET findings (cancers other than lung cancer, analysis according to patients)

Objective of FDG-PET^a^	No. of patients	Modification rate (%) (no. of modified cases)	95 % CI^b^ (two tailed) for modification rate (%)
Breast cancer	126	44.4 (56)	35.6–53.6
Differential diagnosis	4	50.0 (2)	6.8–93.2
Staging	35	25.7 (9)	12.5–43.3
Metastasis/recurrence diagnosis	87	51.7 (45)	40.8–62.6
Colorectal cancer	82	75.6 (62)	64.9–84.4
Staging	13	53.8 (7)	25.1–80.8
Metastasis/recurrence diagnosis	69	79.7 (55)	68.3–88.4
Head/neck cancer	23	65.2 (15)	42.7–83.6
Staging	11	54.5 (6)	23.4–83.3
Metastasis/recurrence diagnosis	12	75.0 (9)	42.8–94.5
Malignant lymphoma	50	70.0 (35)	55.4–82.1
Staging	16	81.3 (13)	54.4–96.0
Metastasis/recurrence diagnosis	34	64.7 (22)	46.5–80.3
Pancreas cancer	20	85.0 (17)	62.1–96.8
Cancer of unknown origin	41	78.0 (32)	62.4–89.4

^a^ Brain tumor (*n* = 3) and malignant melanoma (*n* = 7) eliminated from this consideration, because there was little number of cases

^b^ 95 % confidence interval

### Subanalysis

#### Modification of treatment plan

Major and minor changes in the treatment plan based on FDG-PET findings were analyzed among the 560 patients with cancer divided into subgroups according to the objectives of the FDG-PET examination (differential diagnosis, disease staging, metastasis/recurrence diagnosis, and primary tumor location identification). The results are shown in Table 7. Modifications of the treatment plans based on the FDG-PET findings were made in 55.4 % of patients. Major changes in the treatment plans were in the range 43.3–68.2 % for patients categorized according to the objectives of the FDG-PET examination. The treatment plan was changed in more than 50 % of patients in the differential diagnosis group, the metastasis/recurrence diagnosis group, and the primary location identification group, with such changes occurring in 224 of the 410 patients. In the disease-staging group, the treatment plan was modified in 44.0 % (66/150) of the patients.

**Table 7:** Modification of treatment plan based on FDG-PET findings

	Differential diagnosis	Staging	Metastasis/recurrence diagnosis	Primary tumor location identification	Total
Major change in category of therapy planning	71	44	133	20	268
Major change in modality of therapy	1	20	9	0	30
Major change in goal of therapy	1	1	3	1	6
Minor change in modality of therapy	1	1	4	0	6
No change	33	84	113	20	250
Total	107	150	262	41	560

#### Intensity of treatment

Modifications of the intensity of treatment were analyzed for patients who had been judged as “treatment needed” (*n* = 156) before the FDG-PET examination. These patients were divided into groups according to the objectives of the FDG-PET examination (differential diagnosis, disease staging, metastasis/recurrence diagnosis, and primary tumor location identification). The results are shown in Table 8. For 89 % (139/156) of patients, the intensity of treatment was unchanged after FDG-PET examination. However, the intensity of treatment was increased after FDG-PET examination in 14.3 % (1/7) of the patients in the differential diagnosis group and 8.8 % (9/102) of the patients in the disease-staging group, whereas it was decreased in 7.7 % (3/39) of the patients in the metastasis/recurrence diagnosis group.

**Table 8:** Modification of the intensity of treatment based on FDG-PET findings

	Differential diagnosis	Staging	Metastasis/recurrence diagnosis	Primary tumor location identification	Total
Increased	1	9	1	0	11
Decreased	0	3	3	0	6
Unchanged	6	90	35	8	139
Total	7	102	39	8	156

### Safety

Of the 565 patients who were administered FDG, 100 patients (17.7 %) experienced adverse reactions, including abnormal changes in the laboratory parameters. Frequent adverse reactions were urinary protein positive (15 cases, 2.7 %), urinary occult blood positive (13 cases, 2.3 %), urinary glucose positive (10 cases, 1.8 %), blood pressure increased (10 cases, 1.8 %) and nausea (5 cases, 0.9 %). All adverse reactions were of minimal severity and posed no clinical problems. No serious adverse reactions were noted during this study.

## Discussion

The present study investigated the proportion of management strategies modified after an FDG-PET examination was performed in addition to the existing test plan. The study was performed using a design based on comparison between the pre-test and post-test periods, which is known to involve various possible biases. With this in mind, the following measures were taken to optimize the study design. In this manner, we sought to ensure the reliability of the efficacy evaluation in this study.

- The data center sets the parameters/indicators for the management strategy evaluation in advance, taking into account the status of FDG-PET use at medical facilities and referring to textbooks, published papers, and guidelines relating to each illness. The data center then obtained detailed records of all modifications that were made to the management strategy after the FDG-PET examination, compared with the pre-FDG-PET strategy. In this way, the validity of the evaluation was ensured.
- Individual investigators made a general assessment of the findings based on the results of tests available at the time of entry and filled in the pre-FDG-PET management strategy on the entry sheet, which was then transmitted by facsimile to the data center. The data center then checked the entry sheet received by facsimile and registered the patient. Only then was the FDG for use in that patient delivered. In this way, it was assured that a series of evaluations of the management strategy had indeed been made prior to the FDG-PET examination.
- Individual investigators filled in the post-FDG-PET management strategy on the case report form by the day when the test affecting the decision on management strategy was performed. The filled-in case report forms were then transmitted by facsimile to the data center. This step was intended to eliminate biases between the pre- and post-FDG-PET periods.

For each lung cancer patient, the management strategy was investigated both before and after FDG-PET and the percentage of patients for whom the strategy was modified (management strategy modification rate) was calculated. The management strategy modification rate was 71.6 % (149/208 patients), which was higher than the target modification rate of 25.6 %. We thus judged that the primary endpoint for this study had been verified. In an analysis of the modification rates according to the objectives of the FDG-PET examinations, the modification rate was 88.0 % (73/83 patients) for the differential diagnosis group, 49.3 % (36/73 patients) for the disease-staging group, and 76.9 % (40/52 patients) for the metastasis/recurrence group; each of these rates markedly exceeded the target modification rate (25.6 %).

The target modification rate for this study was based on the modification rate for previous reports evaluating the efficacy of the FDG-PET examinations in patient management [5, 9–24]. The modification rates varied among these previous reports. The differences among the previous reports used for the study design and also between the target modification rate and the modification rate in this study could come from the different populations (e.g., the number of patients by the stage of lung cancer) and so on.

Among the patients enrolled in this study, the pre-FDG-PET judgments made regarding lung cancer and other types of cancer were sometimes “difficult to devise a test plan at present” or “difficult to devise a treatment plan at present”. This uncertainty seems to have contributed to the high strategy modification rate after the FDG-PET examination. The patient eligibility criteria for the present study were similar to the criteria used for coverage under the national health insurance system in Japan as of 2005. This situation probably explains why, among the patients enrolled in this study, there were many for whom devising a test or treatment plan was difficult before the FDG-PET examination.

In the present study, the management strategy modification rate was also within a favorable range (44.4–85.0 %) for cancers other than lung cancer overall (breast cancer, colorectal cancer, head/neck cancer, malignant lymphoma, pancreas cancer and cancer of unknown origin). These results suggest that FDG-PET contributes to the determination of management strategies not only in patients with lung cancer, but also in patients with other types of cancer as well.

According to the National Oncologic PET Registry (NOPR) report [30], major modifications were made to the management strategy in 30.3–39.7 % of patients undergoing a FDG-PET examination for the purposes of diagnosis, initial staging, restaging, or suspected recurrence. In the present study, major modifications of the treatment plan were made in 43.3–68.2 % of the patients who underwent FDG-PET examinations for these same purposes. The NOPR report additionally showed that minor changes in the management strategy were made at a frequency close to that of the major changes. In the present study, on the other hand, the highest frequency of minor changes was 1.5 % (for patients who underwent FDG-PET examinations for the diagnosis of metastasis/recurrence), and major changes were, instead, predominant. The reason why the results of this study differ from the results of the NOPR report was thought to be due to the different populations (the main types of cancer) and study design. Actually, our study design was one of the most objective and accurate evaluation method for strategy modification among previous reports. These results indicate that FDG-PET has a large impact on determining the treatment plan for the types of cancer for which the use of this imaging has been approved in Japan.

Regarding management plans devised before and after FDG-PET, the NOPR report [30] stated that the intensity of treatment was increased in 10 % of all cases and decreased in 22 % after FDG-PET. In the present study, the intensity of treatment was increased in 14.3 and 8.8 % of patients who underwent an FDG-PET examination for a differential diagnosis and disease staging, respectively, and was decreased in 7.7 % of patients who underwent an FDG-PET examination for the diagnosis of metastasis/recurrence. Thus, the intensity of treatment was changed in about 10 % of all patients after the FDG-PET examination. The increase in treatment intensity probably resulted from judgments regarding the need to use additional treatment methods based on the FDG-PET findings, and the reduction in the treatment intensity probably resulted from judgments regarding the feasibility of skipping some treatment methods. Thus, these results suggest that FDG-PET examinations also have an impact on treatment intensity.

The NOPR report [30] stated that it was not possible to judge whether modifications planned after FDG-PET were appropriate or would provide long-term benefits. In the present study, the management strategy modification rate was calculated and analyzed by comparing the pre-FDG-PET strategies and the post-FDG-PET strategies to evaluate step 3 in a hierarchical evaluation model (efficacy for patient management) to assess the efficacy of diagnostic imaging. However, since the present study did not collect data on the relationship between post-FDG-PET treatment and patient outcome, we cannot discuss such a relationship at this time. To resolve these limitations, a study evaluating step 4 of the hierarchical evaluation model (efficacy for promoting patient’s health) is needed. However, such a study will not be easy to implement because it will require a long period to follow-up patient outcomes.

The present study has several limitations. First, the number of patients in several types of cancer was small, which can be explained by the fact only a few patients undergo the FDG-PET examination. Second, this study used several types of PET or PET/CT cameras for the FDG-PET examination. The FDG-PET examinations are better to be performed under some kinds of standardization for PET imaging systems.

## Conclusions

The present study was performed as a multicenter study using a design based on comparisons between pre-test and post-test strategies to evaluate the efficacy of FDG-PET for patient management. The patient management strategies for lung cancer patients were modified after the FDG-PET examinations in 71.6 % of patients analyzed, which was higher than the target modification rate of 25.6 %. Thus, the primary endpoint was verified. There were no serious adverse reactions to the FDG-PET examination, and no concerns were raised from the risk–benefit standpoint. FDG-PET appears to have an impact on decisions regarding the need for additional tests, judging the appropriateness of treatment, and adopting management strategies when dealing with patients encountered during clinical practice whose optimal test plans or treatment plans are difficult to devise.

## Appendix

### Principal investigators and their institutions

Shinsuke Matsuno, Department of Radiology, Takinomiya General Hospital. Nobuo Morioka, Department of Radiology, Matsue Red Cross Hospital. Shuji Adachi, Patient Care Department & Department of Radiology, Hyogo Cancer Center. Mitsuru Koizumi, Department of Nuclear Medicine, Cancer Institute Hospital. Hikaru Seto, Department of Radiology, Toyama University Hospital. Motohisa Kojo, Department of Surgery, Ako Central Hospital. Satoshi Nishioka, Department of Surgery, Ako Hakuho-kai Hospital. Michihiko Nishimura, Department of Internal Medicine and Clinical Laboratory, Koyo Hospital. Hiroshi Yamamoto, Department of Radiology, Sumitomo Hospital.

### Coordinator

Jun Hatazawa, Department of Nuclear Medicine and Tracer Kinetics, Graduate School of Medicine, Osaka University.

### Medical experts

Kazuo Kubota, Department of Radiology, National Center for Global Health and Medicine. Yasushi Takagi, Medical Education Promotion Office, Department of Clinical Pathology, Showa University School of Medicine.
